# C-reactive protein in patients with advanced metastatic renal cell carcinoma: Usefulness in identifying patients most likely to benefit from initial nephrectomy

**DOI:** 10.1186/1471-2407-12-337

**Published:** 2012-08-02

**Authors:** Hiroki Ito, Koichi Shioi, Takayuki Murakami, Akitoshi Takizawa, Futoshi Sano, Takashi Kawahara, Nobuhiko Mizuno, Kazuhide Makiyama, Noboru Nakaigawa, Takeshi Kishida, Takeshi Miura, Yoshinobu Kubota, Masahiro Yao

**Affiliations:** 1 Department of Urology, Yokohama City University Graduate School of Medicine, 3-9 Fukuura, Kanazawa-ku, Yokohama, Kanagawa 236-0004, Japan; 2 Department of Urology, Kanagawa Cancer Center, Yokohama, Japan

**Keywords:** C-reactive protein, Rena cell carcinoma, Prognosis

## Abstract

**Objective:**

C-reactive protein (CRP) is considered a useful serum marker for patients with RCC. However, its clinical utility in advanced metastatic renal cell carcinoma (AM-RCC), particularly in deciding whether to perform nephrectomy at the onset, is not well studied.

**Patients and methods:**

We retrospectively evaluated 181 patients with AM-RCC, including 18 patients underwent potentially curative surgery, 111 underwent cytoreductive nephrectomy, and 52 received medical treatment only. CRP cutoff points were determined by receiver operating characteristic (ROC) curve analysis. Kaplan-Meier and Cox regression analyses were used for survival tests.

**Results:**

ROC analysis suggested that grouping patients according to 3 CRP ranges was a rational model. Patients with highly elevated CRP (≥67.0 mg/L) presented remarkably poor prognosis despite treatment (nephrectomy or medical treatment only). Cox regression models demonstrated that risk factors of overall survival for patients who underwent nephrectomy were the CRP ranges defined in this study (≤18.0 mg/L, >18.0 and <67.0 mg/L, and ≥67.0 mg/L), ECOG PS (0, 1, and ≥2), and number of metastatic organ sites (0–1 and ≥2). The retrospective design is a limitation of this study.

**Conclusion:**

Our study demonstrated that the serum CRP level is a statistically significant prognostic parameter for patients with AM-RCC. The data also indicated that pretreatment serum CRP level provides useful prognostic information that helps in deciding whether to perform initial nephrectomy for patients with AM-RCC.

## Background

Advanced metastatic-renal cell carcinoma (AM-RCC) is associated with a poor prognosis, with the 5-year survival rate being less than 20% for patients presenting with stage IV disease [1,2]. RCC is known to be resistant to conventional chemotherapy and radiation. Cytokine-based immunotherapies, including interferon-alpha (IFN-α) and interleukin-2 (IL-2), elicited limited response in a small subset of AM-RCC cases [3,4]. Over the last few years, novel molecular targeting agents, such as antiangiogenic drugs and mammalian target of rapamycin (mTOR) inhibitors, have been developed, and they have assumed a predominant role in the several treatment options currently available for AM-RCC. On the other hand, surgical interventions, including cytoreductive nephrectomy and/or metastasectomy, have also proven to be beneficial to some extent for patients with AM-RCC, in terms of both survival and quality of life [3-5]. Surgical removal of the majority of tumor burden can be expected to diminish the source of tumor-promoting growth factors or immunosuppressive cytokines, although this has not yet been confirmed [3,4]. Currently, an important concern is that no consensus has been established on the criteria for identifying patients with AM-RCC who are most likely to benefit from nephrectomy and/or metastasectomy performed at the onset [4-7].

C-reactive protein (CRP) is an acute-phase reactant protein exclusively synthesized by hepatocytes. The serum concentration of CRP rises as much as 1000-fold in immediate response to cytokines or chemical mediators released in various pathological conditions, including acute inflammation, infection, tissue or cell necrosis, and some malignancies [7,8]. Several studies have reported that CRP is a useful serum marker for patients with RCC, and elevated serum CRP levels have been shown to be associated with tumor aggressiveness, recurrence, and poor prognosis [8-11]. However, its clinical utility in AM-RCC, particularly in deciding whether to perform surgical interventions at the onset, has not been studied well. We retrospectively investigated the role of CRP as a prognostic marker for patients with AM-RCC. We also explored its usefulness in identifying patients who are most likely to benefit from early nephrectomy.

## Methods

### Patients and treatment

Eligibility for the study was defined as the presence of clinical or pathologic T4, nodal or organ metastatic RCC when the condition was first diagnosed. A total of 181 patients who were treated at our hospitals for AM-RCC between April 1989 and June 2009 were enrolled in the study. The study was conducted in accordance with the principles espoused by the Declaration of Helsinki and all local regulations. The study protocol (#B110901012) was approved by the institutional ethics committee of the Yokohama City University Hospital. Among 181 AM-RCC patients, 18 patients (9.90%) underwent potentially curative surgeries, i.e., radical nephrectomy concomitant with adjacent organ resection and/or total metastasectomy. On the other hand, 111 patients (61.3%) underwent cytoreductive nephrectomy, while the remaining 52 (28.7%) did not undergo nephrectomy and received only medical treatment. Further, 120 of the 129 patients (93.0%) who underwent any kind of nephrectomy (curative or cytoreductive) also received postoperative immunotherapy, including IFN-α and/or IL-2. Among the 52 patients (92.3%) who did not undergo nephrectomy, 42 received immunotherapies. Molecular targeting agents, including sorafenib, sunitinib, and/or everolimus, were administered to 7 patients after 2008. Histopathologic analysis proved that 134/181 (74.0%) patients had clear-cell-RCC, and 174/181 (96.1%) had more than 1 organ metastases (Table 1). At the end of the study period, in April 2011, the follow-up period for all patients ranged from 0.03 to 186 months (median: 28.6 months).

**Table 1:** Clinicopathologic characteristics of patients with advanced metastatic RCC treated with any kind of nephrectomy and no nephrectomy

**Characteristic**	**Any nephrectomy**	**No nephrectomy**	**P**
Number of patients (%)	129 (71.3)	52 (28.7)	
Age, yr			
Median	62	67.5	0.006*
Range	30–81	36–86	
Sex			
Female	33	15	0.780**
Male	96	37	
PS			
0	64	14	<0.001*
1	57	20	
≥2	8	18	
No. of metastatic organ sites			
0(T4N0M0)	6	1	0.295*
1	82	22	
2	17	6	
≥3	24	23	
Metastatic area			
Lung	68	30	0.657**
Bone	46	13	0.227**
Lymph node	31	19	0.089**
Liver	4	7	0.008**
CNS	4	4	0.174**
Others	17	7	0.856*
Median	8.00	9.25	0.008*
Range	2.50–	24.0	
Histopathology			
Clear cell	109	17	
Papillary	4	0	
Chromophobe	1	0	
Collecting-duct	4	0	
Sarcomatoid change	3	5	
Others or not determined	8	30	
Tumor grading (Fuhrman)			0.205*
G1	4	1	
G2	43	4	
G3	57	7	
G4	20	6	
X		5	34

*Mann-Whitney U test; **Chi-square test.

### Clinical and laboratory assessment

All patients were staged according to the 2002 TNM classification system [12]. We collected data on the following clinicopathologic factors from the medical records of the patients: patient age; sex; Eastern Cooperative Oncology Group performance status (ECOG PS); maximum tumor diameter; number of metastatic organ sites; and levels of hemoglobin (Hb), lactate dehydrogenase (LDH), alkaline phosphatase (ALP), corrected calcium, and CRP when a patient was first seen. The Hb level was categorized using a cutoff value of the lower normal limit, and LDH and ALP levels, using a cutoff value of 1.5× the normal limit; corrected calcium was considered normal up to 10 mg/dL, as reported previously [1,12,13]. Maximum tumor diameter was divided into two groups using median value.

### Statistical analysis

The overall survival (OS) period was calculated from the date of nephrectomy to death or last follow-up. For patients who did not undergo nephrectomy, the survival period was measured from the first diagnosis of RCC to death or last follow-up. The differences between groups were examined using the Mann–Whitney *U* or chi-square test depending on the data set.

We determined the cutoff point of CRP according to the sensitivity and specificity levels derived from the receiver operating characteristic (ROC) curve plotted using death before median survival period in the patient cohort that underwent nephrectomy. Survival probabilities were estimated using the Kaplan-Meier method, and the resultant curves were statistically tested by the log-rank method. Cox proportional hazards model was used for univariate and multivariate analyses. Significance was set at *P* < 0.10, which was used as the criterion for determining variable entry and removal from the multivariate analysis. All data were analyzed using the SPSS software package (SPSS, Chicago, Illinois). All statistical tests were two-sided and were considered to be statistically significant for *P* < 0*.*05.

## Results

### Patient characteristics

We initially tried to figure out what kind of clinical factors might influence on the selection of initial nephrectomy for AM-RCC patient although the study was retrospective, non-randomized observational setting. We therefore compared the clinicopathologic characteristics of patients who underwent curative or cytoreductive nephrectomy with those of patients who did not undergo nephrectomy. As expected, older patient age (*P* = 0.003), larger tumor size (*P* = 0.004), and poorer ECOG PS (*P* < 0.001) were observed in the non-nephrectomy group compared to the nephrectomy group (Table 1). With respect to the metastatic organ sites, only the liver showed a higher incidence in the non-nephrectomy group than in the other group (*P* = 0.008); no apparent intergroup differences were noted in the incidence of metastasis at any other organ sites (*P* = 0.295; Table 1).

### Survival distribution

**Figure 1:**
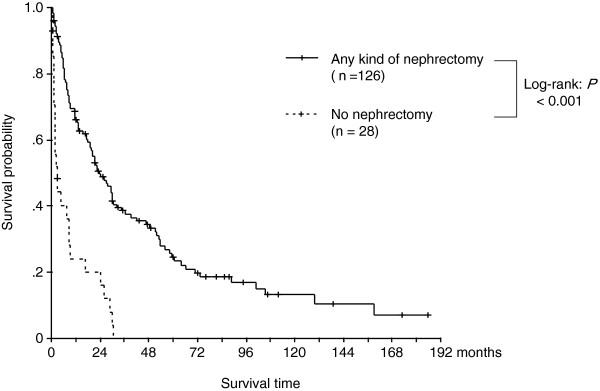
Kaplan-Meier overall survival probability curves for the 2 treatment groups (patients who underwent nephrectomy and those who did not).

Next, we analyzed the overall survival period for patients who underwent nephrectomy and those who did not. As reported previously, nephrectomy provided strong survival advantage for AM-RCC patients. The median survival for the 129 patients who underwent nephrectomy and the 52 patients who did not undergo nephrectomy was 23.9 months and 2.80 months (*P* < 0.001), respectively (Figure 1).

### C-reactive protein and patient survival

**Figure 2:**
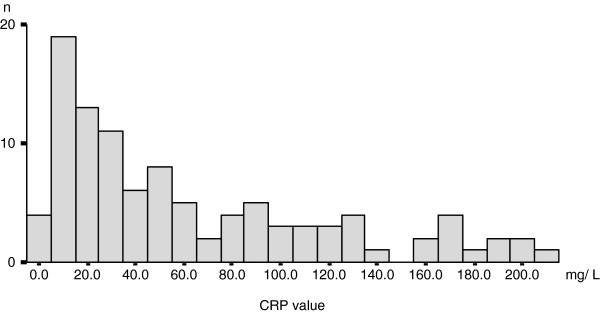
Histogram of elevated CRP value in patients with AM-RCC. Among 143 patients with AM-RCC, 103 (72.0%) exhibited abnormal CRP values (>3.00 mg/L) at initial presentation; the graph was plotted for these values.

From the medical records, we collected data regarding CRP levels at the onset in 143/181 AM-RCC patients. The patients exhibited varying serum CRP levels, ranging from undetectable to 212 mg/L, and 103/143 (72.0%) patients had abnormal CRP values (over 3.00 mg/L; Figure 2).

**Figure 3:**
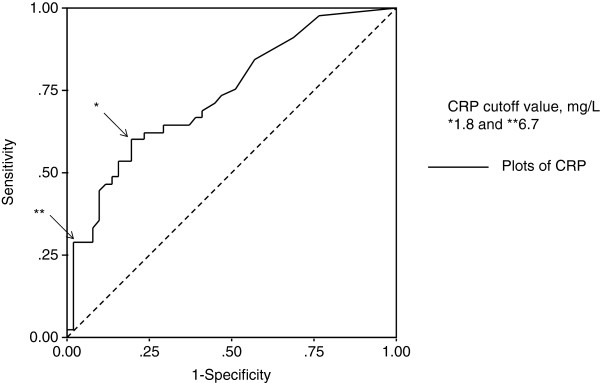
ROC curve of CRP values for overall rate of death before the median survival period. Cohort of patients with AM-RCC who underwent nephrectomy (n = 95; median survival = 25.7 months) was analyzed.

**Figure 4:**
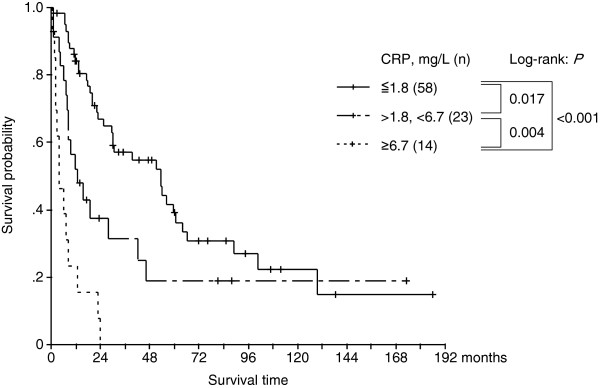
Kaplan-Meier overall survival probability curves according to the 3 CRP-defined groups of patients who underwent nephrectomy (n = 95).

We determined the CRP cutoff point by means of ROC analysis for the cohort of patients who underwent nephrectomy (n = 95). From the ROC analysis, we found 2 reasonable CRP cutoff points, 18.0 and 67.0 mg/L (Figure 3); accordingly, we classified the patients into normal/mildly elevated CRP (≤18.0 mg/L), elevated CRP (18.0–67.0 mg/L), and highly elevated CRP (≥67.0 mg/L) groups. Kaplan-Meier analyses revealed statistically significant differences between these 3 groups, and the median overall survival periods were 53.2, 12.6, and 4.20 months in the normal/mildly elevated CRP, elevated CRP, and highly elevated CRP groups, respectively (Figure 4).

**Figure 5:**
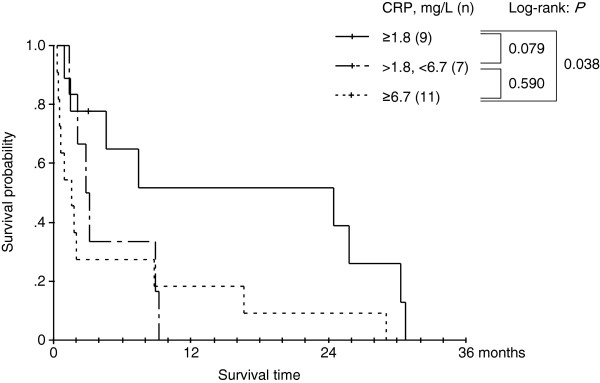
Kaplan-Meier overall survival probability curves according to the 3 CRP-defined groups of patients who did not undergo nephrectomy (n = 27).

We next applied the same CRP cutoff and grouping system to the AM-RCC cohort of patients who did not undergo nephrectomy. We found that the 3 CRP levels defined were again correlated with patient survival and that patients with normal/mildly elevated CRP levels clearly showed longer survival periods (Figure 5). The median overall survival periods were 24.4, 2.83, and 1.54 months in the normal/mildly elevated CRP, elevated CRP, and highly elevated CRP groups, respectively.

Since patients with highly elevated (≥67.0 mg/L) levels of CRP showed poor prognosis despite the treatment procedures, i.e., nephrectomy or medical treatment only (median OS: 4.20 and 1.54 months, respectively), we compared these 2 groups. Kaplan-Meier analysis showed that the 2 groups did not differ statistically (log-rank: *P* = 0.703). Although the 2 groups differed slightly with respect to patient characteristics and backgrounds, the data suggested that initial nephrectomy did not seem to provide substantial survival advantages for patients with highly elevated CRP levels.

### CRP as an independent prognostic parameter for AM-RCC

Lastly, we tested the association of patient survival with CRP values against that with other established clinical and biochemical parameters, including the Memorial Sloan-Kettering Cancer Center (MSKCC) risk factors [1,2]. Survival analysis was performed for 81 patients who underwent nephrectomy and for whom the complete clinical and biochemical data were available. Cox univariate analyses demonstrated that several clinicopathologic parameters, including age; ECOG PS; number of metastatic organ sites; and levels of Hb, ALP, and CRP, showed a statistically significant association with OS (Table 2). On the other hand, neither LDH (*P* = 0.567) nor corrected calcium (*P* = 0.404) levels were correlated with survival lengths. Further Cox multivariate analysis demonstrated that ECOG PS, number of metastatic organ sites, and the CRP levels defined in this study remained statistically significant independent parameters for survival (Table 2).

**Table 2:** Univariate and multivariate Cox regression analysis of overall survival among patients AM-RCC who underwent nephrectomy of any kind (n = 81)

**Parameter**	**Univariate**				**Multivariate**		
	**n**	**P**	**HR**	**95% CI**	**P**	**HR**	**95% CI**
Age, yr							
<62.0	39		1.00			1.00	
≥62.0	42	0.026	1.87	1.08−3.26	0.177	1.51	0.083−2.76
Sex							
Female	25		1.00			X	
Male	56	0.840	0.94	0.53-1.68			
ECOG PS							
0	44	1.00				1.00	
1	32	0.001	2.54	1.45-4.34	0.012	2.22	1.19-4.16
≥2	5	0.000	7.89	2.56-24.3	0.022	4.18	1.23-14.3
Hb							
≥Lower normal limit	45		1.00			1.00	
<Lower normal limit	36	0.047	1.72	1.01-2.92	0.862	1.06	0.58-1.93
LDH							
≦Normal limit × 1.5	51		1.00			x	
>Normal limit × 1.5	30	0.567	1.17	0.69-2.00			
ALP							
≦Normal limit × 1.5	78		1.00			1.00	
>Normal limit × 1.5	3	0.026	3.92	1.18-13.0	0.510	0.60	0.13-2.76
Corrected Ca, mg/dL							
<10.0	50		1.00			x	
≥10.0	31	0.404	0.79	0.45-138			
Max. tumor diameter, cm							
<8.0	41		1.00			x	
≥8.0	40	0.381	0.78	0.45-1.35			
No. of metastatic organ sites							
0,1	35		1.00				
≥2	46	0.054	1.72	0.99-2.99	0.011	2.33	1.21-4.48
CRP, mg/L							
≦18.0	49		1.00			1.00	
>18.0, <67.	20	0.057	1.86	0.98-3.54	0.036	2.06	1.05-4.05
0 ≥ 67.0	12	0.000	6.66	3.16-14.01	0.001	5.85	2.07-16.56

*X, excluded from the multivariate analysis.

## Discussion

CRP is a standardized and widely used serum indicator of acute-phase response in conditions such as acute inflammations, infections, tissue or organ necrosis, and malignancies [14]. Some types of RCCs can induce systemic inflammations by expressing various cytokines such as IL-1, tumor necrosis factor, and mostly IL-6 [15]. In vitro experimental studies showed some renal tumors themselves are actually capable of producing IL-6 [15]. Furthermore, RCC, especially of the aggressive phenotype, is often accompanied by tumor necrosis [16]. Therefore, tumor status and aggressiveness can be directly reflected by the serum CRP levels of patients with AM-RCC. Additionally, in case of other malignancies, CRP had been found to inhibit apoptosis of carcinoma cells, thereby directly regulating tumor cell growth and survival [17]. In fact, we often encounter cases of AM-RCC in which the patient’s CRP level fluctuates in accordance with disease control and/or progression. Several studies have also indicated that elevated CRP is a poor prognostic indicator for RCC [9-11,18,19]. However, almost all the CRP cutoff points reported previously have been single values. These cutoff points range from 1.0 to 10.0 mg/L and vary widely from study to study, despite the fact that in the majority of studies, the cutoff point was defined on the basis of the normal values [9-11,18,19]. In the current study, we found that over 70% of the patients with AM-RCC showed abnormal CRP values, with a relatively wide dynamic range of up to 200 mg/L (Figure 2). In fact, when we initially applied previously reported cutoff points to our patient cohort, we were unable to define any CRP-based grouping system that could afford a rational assessment of survival risk (data not shown). In this study, we initially determined the 2 CRP cutoff points 18.0 and 67.0 mg/L in the nephrectomy patient cohort by means of ROC analysis. We then found that the CRP 3 grouping was a more suitable model for the risk stratification of AM-RCC patients because it was also applicable for patients who did not undergo nephrectomy (Figure 5).

A number of prognostic parameters have been studied for patients with localized, metastatic, or all-stage RCC [1,2,16,19-27]. Among the parameters defined for AM-RCC, the most well-established and validated ones are Memorial Sloan-Kettering Cancer Center (MSKCC) risk factors [1] and predictors of short survival proposed by the NCCN practice guidelines [12]. In these models, however, CRP has not been enlisted as a significant factor. In our Cox models, we found that the risk factors associated with shorter survivals were high CRP level, low ECOG PS, and high number of metastatic organ sites (Table 2). On the other hand, Hb and ALP levels were not found to be independent parameters. Moreover, sex, maximum tumor diameter, LDH and corrected calcium levels did not appear to be statistically significant factors even in the univariate analyses. Thus, our results were considerably different from the recommendations of the MSKCC and NCCN guidelines [1,12]. One of the major reasons for this discrepancy should be the treatment modalities. In our regression model, all patients underwent nephrectomy as the initial treatment. On the other hand, the prognostic factors defined in the MSKCC or NCCN guidelines were originally elucidated from patients receiving non-surgical medical treatments. Another possible reason might be the racial differences. Enrolled patients in our study were Japanese Asian populations. Naito S, et al. suggested that Japanese patients with AM-RCC showed better prognosis than non-Asian cohort [22]. Such racial differences might affect the biological character of tumors, leading to differences in present report. On the basis of our data, we recommend that for patients with AM-RCC who are being considered for nephrectomy as the initial treatment option, more importance should be given to the CRP levels, ECOG PS, and the number of metastatic organ sites than other patient characteristics, such as ALP, Hb, LDH, and serum Ca levels.

Reports indicate that surgical complications occur in 20–22 % of patients with AM-RCC, and therefore, surgical approaches should not be adopted indiscriminately [5,6]. Our data revealed that the patient group with highly elevated CRP (≥67.0 mg/L) showed very poor prognosis despite of treatment (median OS: 4.20 months in the nephrectomy group and 1.54 months in the non-nephrectomy group) (Figures 4 and 5). In this patient subgroup, the indications of initial surgical intervention should be assessed very carefully. On the other hand, the normal/mildly elevated CRP (≤18.0 mg/L) group showed relatively good prognosis even for patients who did not undergo nephrectomy (median OS: 24.2 months; Figure 5). Our data also suggested that the initial nephrectomy seemed to provide considerable survival advantages to patients with normal/mildly elevated and presumably elevated CRP levels (Figure 4). Thus, surgical approaches appear to be beneficial for these patient groups although other parameters need to be evaluated to validate this finding.

The majority of the patients analyzed in this study belonged to the so-called cytokine era. Currently, a number of treatment options, such as surgery, immunotherapy, and a series of molecular targeting agents, are available for patients with AM-RCC. It has been well-demonstrated that both MSKCC risk factors and NCCN guideline parameters, which were originally established in the cytokine era, are useful even in the molecular targeting era. Therefore, we expect that the current CRP grouping model will be important in the risk classification of patients with AM-RCC, even in the current molecular targeting era. Further prospective studies are now required to validate our findings.

There are inherent limitations to this study. Because of its retrospective design, the confounding factors and measurement bias cannot be reduced as much as they could in prospective, randomized studies. Especially, there is clearly a selection bias in any comparison between the nephrectomy and non-nephrectomy groups since the latter were clearly more advanced/unresectable/non-surgical candidates. However, despite of no clear criteria whether surgery was performed or not, Table 1 revealed significant differeneces; patient age, tumor size, ECOG PS and liver metastasis. That suggested that these parameters had impacts on our decision making. We believed that might be also useful information for clinical practice [3,4,6].

## Conclusions

Our study demonstrated that the serum CRP level is a statistically significant prognostic parameter for patients with AM-RCC. Risk factors of OS were CRP (≤18.0 mg/L, >18.0 and <67.0 mg/L, and ≥67.0 mg/L), ECOG PS (0, 1, and ≥2), number of metastatic organ sites (0–1 and ≥2). The study also revealed that patients with highly elevated CRP levels (≥67.0 mg/L) had a considerably poor prognosis despite treatment. The data indicated that pretreatment serum CRP level could be one of the most reliable prognostic factors that can help decide whether to perform surgical procedures for patients with AM-RCC.

## Abbreviations

AM-RCC, Advanced Metastatic Renal Cell Carcinoma; CRP, C-Reactive Protein; ECOG PS, Eastern Cooperative Oncology Group Performance Status; Hb, Hemoglobin; LDH, Lactate dehydrogenase; ALP, Alkaline Phosphatase; ROC, Receiver Operating Characteristic.

## Competing interests

The author declare that they have no conflict of interests

## Authors’ contributions

All authors participated in the design and conduct of the study. All authors reviewed and approved the final version of the manuscript.

## Pre-publication history

The pre-publication history for this paper can be accessed here:

http://www.biomedcentral.com/1471-2407/12/337/prepub
